# Sunglasses for Sunlight: Considerations on New Treatment Opportunities for Refractory Colorectal Cancer

**DOI:** 10.3390/cancers16071348

**Published:** 2024-03-29

**Authors:** Alberto Zaniboni

**Affiliations:** Oncologia Medica, Fondazione Poliambulanza, 25124 Brescia, Italy; azaniboni@alice.it; Tel.: +39-3356095439

In 2023, two seminal studies were disseminated that significantly augmented the pharmacological armamentarium for the treatment of refractory metastatic colorectal carcinoma (MCRC). The Sunlight investigation undertook a comparative analysis of TAS-102 against a combination of TAS-102 and bevacizumab [1] Conversely, the Fresco 2 study scrutinized fruquintinib, an efficacious oral inhibitor targeting VEGFR 1-2-3, against a placebo in a pretreated MCRC population that had previously undergone treatment with TAS-102, regorafenib, or a combination of both [2]. The findings from both studies were positive: in the Sunlight trial, an open label study with about 3% of patients recruited in the USA, the combination of TAS-102 and bevacizumab exhibited superior effectiveness compared to TAS-102 alone. Specifically, the observed results showed 10.8 months versus 7.5 months for overall survival (OS) (HR 0.61), 5.6 months versus 2.4 months for progression-free survival (PFS) (HR 0.44), and a median time to performance status deterioration of 9.3 months versus 6.3 months (HR 0.54), with a toxicity profile deemed acceptable. The global medical oncology community greeted these outcomes with enthusiasm, recognizing the clinical importance of the addition of bevacizumab, which had not previously shown such results beyond the second line of treatment. However, it was somewhat disappointing to observe that TAS + bevacizumab did not outperform capecitabine + bevacizumab in first-line treatment in the Solstice study [3]. Similarly positive were the findings from the Fresco 2 study, in which fruquintinib demonstrated superiority over the placebo for overall survival (OS), 7.4 months versus 4.8 months (HR 0.66), and for progression-free survival (PFS), 3.7 months versus 1.8 months (HR 0.32), in a more heavily pretreated population of patients when compared with the Sunlight patients’ characteristics. Hypertension and fatigue emerged as the primary side effects. In our clinical practice, what criteria should be considered when comparing and selecting between these two treatment options?

If we scrutinize the patient characteristics within the Sunlight study, as per the latest updates presented at the ESMO 2023 congress [4], it becomes apparent that, on the whole, these patients cannot be definitively categorized as heavily pretreated, particularly in the context of anti-angiogenic therapy. Specifically, only 76% of the subjects had received prior anti-VEGF treatment, and a mere 20% had undergone two rounds of anti-angiogenic therapy before the administration of TAS + beva. In this latter subgroup, the Hazard Ratio (HR) for overall survival (OS) stands at 0.76 (0.52–1.10) according to a post hoc analysis. It is noteworthy that the study and the ESMO update do not provide information on the utilization of an alternative anti-angiogenic agent, such as aflibercept or ramucirumab, after bevacizumab. Hence, one could hypothesize that the 20% of patients subjected to two lines of anti-angiogenic therapy predominantly adhered to a strategy involving continued use of bevacizumab beyond progression. Given the perceived importance of sustaining uninterrupted angiogenesis inhibition, especially in patients with Ras mutations, the current consensus, at least for the initial and subsequent treatment lines, involves contemplating a sequential approach, such as the following:

Administration of FOLFOX + bevacizumab followed by FOLFIRI + bevacizumab or its reverse counterpart [5]. Alternatively, FOLFOX + bevacizumab followed by FOLFIRI + aflibercept (or ramucirumab, if reimbursed) [6] represents a therapeutic strategy for a specific patient subgroup. However, it is notable that such patients are inadequately represented in the Sunlight study. The following question arises: will the notable benefits observed with TAS-102 + beva be equally applicable to this patient demographic, commonly encountered in European and North American outpatient settings? In the case of fruquintinib, a noteworthy critique of the Fresco-2 study pertains to the inclusion of a placebo arm. This stands in contrast to one of the primary strengths of the Sunlight trial, where the presence of a control arm (TAS-102) is deemed the standard of care in this context. Fresco-2 may likely represent the concluding study of its kind to incorporate a placebo arm. The dilemma of selecting between TAS-102 + bevacizumab and fruquintinib persists. It is pertinent to note that the latest version of the NCCN guidelines (COL-D 4 OF 11, Version 1.2024) uniformly categorizes these drugs and regorafenib without suggesting a preferred sequence. My personal perspective is that the Sunlight regimen may not be suitable for all comers. Instead, careful attention should be given to the type of prior anti-angiogenic treatment received (duration, tolerance, outcomes), as well as patient preferences (commitment to 2/3 monthly intravenous bevacizumab administrations versus a completely oral treatment). Sunlight could be an excellent strategy, for instance, for patients with Ras wild-type tumors who have typically undergone a single line of prior anti-angiogenic therapy, either before or after anti-EGFR treatment depending on tumor location. Additionally, the regimen might prove optimal for patients who have not extensively utilized anti-angiogenic treatment in the first and second lines; this is a demographic seemingly encompassing a substantial portion of those enrolled in the Sunlight study. Fruquintinib could be considered for patients who have derived prolonged benefits from prior anti-angiogenic treatment and/or prefer an exclusively oral treatment approach. The pharmacological management of refractory metastatic colorectal cancer (MCRC) with conventional therapeutics is undergoing enrichment with the introduction of novel molecules and strategic approaches, thereby expanding the therapeutic choices available to clinicians and patients alike. Emerging molecular targets appear poised for application in earlier treatment lines (HER-2, KRAS G12C, Braf-mutated, MSI, etc.), which is distinct from the dichotomy observed in the Sunlight/Fresco paradigm. Furthermore, it is imperative to consistently evaluate alternatives, such as regorafenib, anti-EGFR rechallenge, intermittent chemotherapy, locoregional interventions, and the potential initiation of patients into novel experimental trials [7]. In conclusion, the Sunlight study is duly recognized as an advancement in MCRC treatment options. Nevertheless, the optimization of treatment outcomes lies in the highest attainable degree of personalization, characterized by the strategic selection of sequential approaches in collaboration with patients.

## Abbreviations

The following abbreviations are used in this manuscript:

5-FU	5-Fluorouraci
BRAF - B	B-Raf Proto-oncogen
EGFR	Epiderma Growth Factor Receptor
ESMO	EUropean Society of Oncology
FOLFIRI	5-FU, Leucovorin, Irinotecan
FOLFOX	5-FU, Leucovorin, Oxaliplatin
HER-2	Human Epidermal Receptor 2
HR	Hazard Ratio
KRAS	Kirsten Rat Sarcoma Viral Oncogene Homolog
MCRC	metastatic colorectal cancer
MSI	Microsatellite Instability
NCCN	National Comprehensive Cancer Network
OS	overall survival
PFS	progression-free survival
TAS-102	Trifluridine/Tipiracil
VEGFR	Vascular ENdothelial Growth Factor Receptor
